# Voltammetric determination of sumatriptan in the presence of naproxen using a modified screen printed electrode

**DOI:** 10.5599/admet.2635

**Published:** 2025-02-19

**Authors:** Hazim Saad Jabbar Al-Maliki, Sudad Jasim Mohammed, Adil Turki Al-Musawi, Aliaa Saadoon Abdul- Razaq Al-Faraji, Mazin Hadi Kzar, Abdul Amir H. Kadhum, Huda Hadi Nameh, Raed Muslim Mhaibes

**Affiliations:** 1 Department of Basic Sciences, College of Dentistry, University of Basrah, Iraq; 2 Market Research and Consumer Protection Center, University of Baghdad, Iraq; 3 College of Physical Education and Sport Sciences, Al-Mustaqbal University, 51001 Hillah, Babil-Iraq; 4 College of Medicine, University of Al-Ameed, Karbala, Iraq; 5 College of Pharmacy, University of Hilla, Babylon, Iraq; 6 Department of Biochemistry, College of Medicine, Misan University, Misan Iraq

**Keywords:** Simultaneous determination, real sample analysis, nanomaterials, drug analysis

## Abstract

**Background and purpose:**

Sumatriptan is used to alleviate symptoms of migraine headaches, particularly during acute attacks. Naproxen is a medication that provides relief from pain, inflammation, and fever. Therefore, determination of them is important.

**Experimental approach:**

In the present work, CoMoO_4_ nanosheets were synthesized in a basic and easy way. A screen-printed graphite electrode's surface was altered using the as-prepared CoMoO_4_ nanosheets' high electroactivity to create a CoMoO_4_ nanosheets-modified screen-printed electrode (CoMoO_4_ NSs-SPE), which was then employed for sumatriptan's electrochemical oxidation. Due to the superior electron transfer characteristics and catalytic activity of the produced CoMoO_4_ nanosheets, the results demonstrated a notable improvement in sumatriptan's current responses. This study examined the electrochemical behavior of sumatriptan on the CoMoO_4_ NSs-SPE utilizing a number of methods, including as chronoamperometry, cyclic voltammetry, and differential pulse voltammetry (DPV).

**Key results:**

With a high sensitivity of 0.0718 μA/μM and a good correlation value of 0.9998, a linear calibration curve was obtained over a broad concentration range of 0.02-600.0 μM, suggesting a strong linear connection between the concentration and the response. Based on a signal-to-noise ratio of 3, the limit of detection for sumatriptan was determined to be 0.01 μM, suggesting a high degree of sensitivity for the detection technique. DPV results showed that the CoMoO_4_ nanosheets-modified screen-printed electrode (CoMoO_4_ NSs-SPE) could detect naproxen and sumatriptan at the same time.

**Conclusion:**

The created sensor's usefulness and efficacy in real-world applications were demonstrated when it was successfully used to identify the target analytes in actual samples.

## Introduction

Sumatriptan is a medication that belongs to the tryptamine class and is widely used to alleviate symptoms of migraine headaches, particularly during acute attacks [1]. Sumatriptan works by activating specific serotonin receptors, known as 5-HT1D and 5-HT1B, which helps to reduce inflammation in blood vessels and relieve migraine symptoms [2]. When a 50 mg sumatriptan tablet is taken orally, it reaches its highest concentration in the blood, approximately 33.21 ng/mL, within 1 hour and 8 minutes. The medication is then gradually eliminated from the body, with a half-life of approximately 2 hours and 58 minutes [3].

It's notable that taking high doses of sumatriptan, specifically 200 mg per day, can potentially cause a rare condition called sulfhemoglobinemia. This condition occurs when sulfur binds to hemoglobin molecules, resulting in a change in blood color from red to a greenish-black hue [4]. Given the biological significance of sumatriptan, considerable research efforts have been focused on developing methods to accurately detect and measure this compound in various biological samples. Among several analytical methods, liquid chromatography is one frequently used for sumatriptan detection by most analysts. Different liquid chromatographic methods (high-performance liquid chromatography [5], ultra-performance liquid chromatography (UPLC) [6], high-performance thin-layer chromatography [7]) have already been implemented and used for sumatriptan assay. Liquid chromatography is not the only analytical method that has been evaluated for the determination of sumatriptan, including micellar electrokinetic chromatography [8], conductometry [9], UV-Vis absorbance spectrophotometry and fluorescence spectroscopy to be able to address its measurement by other routes. Although a few of them have demonstrated good sensitivity, the cheap alternative most often confuses durability for lengthy and laborious procedures, costly utility or sensitive standards, complexing agents, or even hazardous solvents hampering practical usage. As a result, there is a pressing need for the development of new methods that can accurately determine sumatriptan in a sensitive, cost-effective, rapid, and straightforward manner, overcoming the limitations of existing approaches. Fortunately, electrochemical sensors have emerged as a promising solution, as previous research has shown that sumatriptan exhibits electrochemical activity, making it a suitable candidate for detection using this approach, which can offer the desired combination of sensitivity, cost-effectiveness, speed, and simplicity. In line with this, researchers have made efforts to develop electrochemical sensing platforms specifically designed for the sensitive detection of sumatriptan, aiming to harness the potential of electrochemical sensors for accurate and efficient analysis [12].

Naproxen is a medication that provides relief from pain, inflammation, and fever and is commonly used to treat various conditions, including rheumatoid arthritis, osteoarthritis, gout, and migraine, as well as other inflammatory disorders. Additionally, naproxen has been found to be effective in alleviating pain associated with orthopedic surgery, as well as relieving muscle cramps and stiffness in certain cases [13]. Naproxen should be used with caution in certain patient populations, including the elderly with kidney problems, as well as those with conditions such as ear duct inflammation, hemophilia, platelet coagulation disorders, and gastrointestinal bleeding due to potential increased risk of adverse effects [13]. Various analytical methods have been established for the detection and quantification of naproxen, catering to different needs and applications. These methods encompass a range of techniques, including capillary electrophoresis [14], spectrophotometry [15], fluorometry [16], spectrofluorometry [17], chemiluminescence [18], high-performance liquid chromatography [19], and electrochemical methods [20-23] each offering distinct advantages and applications for the analysis of naproxen. Electrochemical techniques have gained significant attention for the determination of naproxen due to their simplicity, cost-effectiveness, and high precision, making them a promising approach for accurate and reliable analysis [23].

The use of conventional electrodes for the electrochemical measurement of pharmaceutical and biological compounds, including naproxen, was limited due to the high overvoltage required. To overcome this challenge, researchers have developed modified electrodes incorporating nanoparticles, improving the sensitivity and efficiency of electrochemical detection. The unique properties of nanostructure materials, which arise from their extremely small size, make them distinct from their bulk counterparts, exhibiting enhanced physical and chemical characteristics that can be leveraged for various applications, including electrochemical sensing. The distinctive properties of nanomaterials make them an attractive tool for advancing and refining analytical techniques, enabling the development of more sensitive, selective, and efficient methods for detecting and quantifying various substances, including pharmaceutical compounds like naproxen [24-29].

In recent years, printing electrode technologies have been rapidly advancing, emerging as a cutting-edge approach for the fabrication of diverse electronic devices, offering a promising solution for the development of innovative and versatile electronic equipment [30]. Printed electrodes have found widespread applications in various fields, including energy storage and conversion, electronic displays, and smart electronic devices, due to their versatility, flexibility, and potential for cost-effective and scalable production [30]. Recent efforts in the field of printed electrode technology have paved the way for the development of facile integrated electrodes that can be directly printed onto electrochemical sensing applications. Besides, many ways for printed electrode fabrication have also been developed by researchers, *e.g.* inkjet printing, 3D printing and screen printing[31,32], raising the hope to produce high-quality electrodes with electrochemical sensing applications. Recent printed electrode technology has been developed to replace conventional solid electrodes in a variety of electrochemical sensing applications and sensors (printable electrode sensors). With this, the detection speed will be very fast, and it will be beneficial for the fabrication of cost-effective devices and their transformable nature, given their advantages; they are the most interesting alternatives to on-chip electrodes [33,34].

CoMoO_4_ nanosheets were used in this work as a modifying agent to alter a screen-printed electrode's (SPE) surface. The electrochemical behavior of sumatriptan was fully examined utilizing a variety of electrochemical methods, such as cyclic voltammetry (CV), chronoamperometry, and differential pulse voltammetry (DPV) after the SPE was modified with CoMoO_4_ nanosheets. Under ideal experimental circumstances, the suggested CoMoO_4_ nanosheet-modified SPE demonstrated remarkable catalytic activity towards sumatriptan. Additionally, utilizing differential pulse voltammetry (DPV), the CoMoO_4_ NSs/SPE sensor was effectively used to determine sumatriptan and naproxen simultaneously. Different oxidation peaks were seen for both substances. Furthermore, the established method's practical applicability and dependability in real-world applications were demonstrated when it was successfully used to accurately and reliably detect naproxen and sumatriptan in actual pharmaceutical samples.

## Experimental

### Apparatus and chemicals

An Autolab potentiostat/galvanostat (PGSTAT 12N) from Eco Chemie, Netherlands, was used for electrochemical experiments. A three-electrode setup including a graphite working electrode (WE), a graphite auxiliary electrode (AE), and a silver pseudo-reference electrode (RE) was used in the screen-printed electrodes (SPEs) manufactured by DropSens (DRP-110, Spain). A Metrohm 710 pH meter was used to measure the pH. Every solution was made from scratch with double-distilled water. All analytical-grade reagents, including naproxen and samaritan, came from Merck (Darmstadt, Germany). Orthophosphoric acid and its salts purchased from Merck (Darmstadt, Germany) were used to make the buffer solutions.

### Synthesis of CoMoO_4_ nanostructures

The following is how the CoMoO_4_ nanostructures were created: First, 30 mL of ethanol and 25 mL of distilled water were used to dissolve 0.25 g of CoCl_2_.6H_2_O and 0.2 g of (NH_4_)_6_Mo_7_O_24_×4H_2_O, which were then agitated for 15 minutes. After that, the solution was put into an autoclave. The solvo/hydrothermal reaction was carried out by placing the autoclave in an oven set to 180 °C for ten hours. The obtained sample was cooled to ambient temperature and then repeatedly cleaned with ethanol and distilled water before being dried for 15 hours at 70 °C. It was then calcined at 400 °C for two hours.

### Preparation of CoMoO_4_ nanosheets/SPE electrode

2 milligrams of CoMoO_4_ nanosheets (NSs) were dispersed in 1 milliliter of ultra-pure water through 15 minutes of dispersion. Subsequently, the working electrode was fabricated by casting 5 microliters of the CoMoO_4_ NSs suspension onto it. The modified electrode was then obtained by allowing the solvent to evaporate. The CoMoO_4_ NSs / SPE was formed after the evaporation of the solvent.

### Preparation of real samples

In order to examine sumatriptan in actual samples, ten pills were broken up and combined, and then 100 mg of the powder was ultrasonically dissolved in 50 mL of water. A 25 mL volumetric flask was then filled to the brim with phosphate-buffered saline (PBS) at pH 7.0 after the resultant solution had been diluted to different concentrations. The examination of these produced samples was carried out using the modified electrode.

To analyze naproxen in real samples, ten tablets were crushed and mixed together, and then 1000 mg of the powder was dissolved in 50 mL of water using ultrasonication. The resulting solution was then diluted to various concentrations and transferred to a 25 mL volumetric flask, filled to the mark with phosphate-buffered saline (PBS) at pH 7.0. The modified electrode was used to perform the analysis on these prepared samples.

## Results and discussion

### Characterization of CoMoO_4_ nanosheets

**Figure 1.**
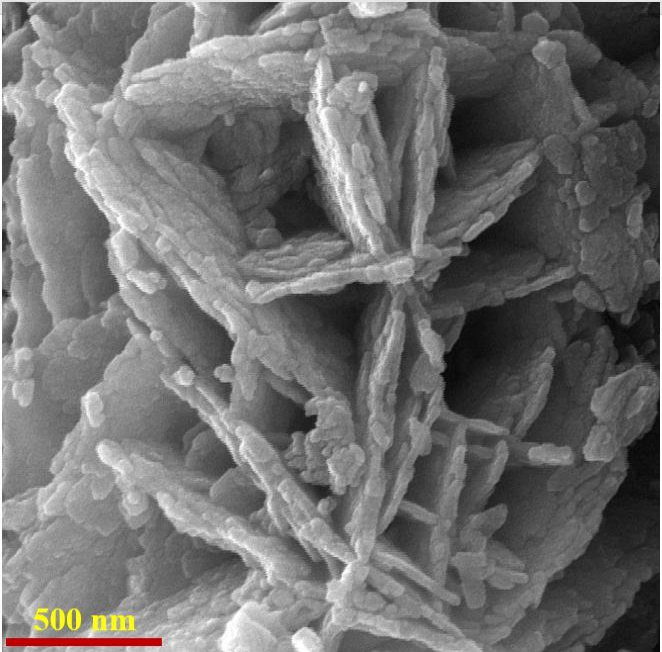
FE-SEM image of CoMoO_4_ nanosheets

The surface morphology of the as-synthesized CoMoO_4_ nanosheets was obtained using FE-SEM. The FE-SEM image of CoMoO_4_ nanosheets is shown in Figure 1.

### Cyclic voltammetric study of sumatriptan oxidation

The pH of the solution plays a crucial role in influencing the electrochemical performance of the CoMoO_4_ NSs-modified SPE. To assess this effect, we systematically recorded the peak current and peak potential of the CoMoO_4_ NSs-modified SPE in the presence of 200.0 μM sumatriptan within a 0.1 M phosphate buffer solution (PBS) at varying pH levels (2.0-9.0) using a scan rate of 50 mV/s. The results demonstrated that the CoMoO_4_ NSs-modified SPE exhibited optimal electrocatalytic activity at a pH of 7.0, indicating that this neutral pH condition is most favorable for the electrochemical reactions involved. Therefore, we proceeded with further electrochemical investigations using PBS at pH 7.0.

**Figure 2.**
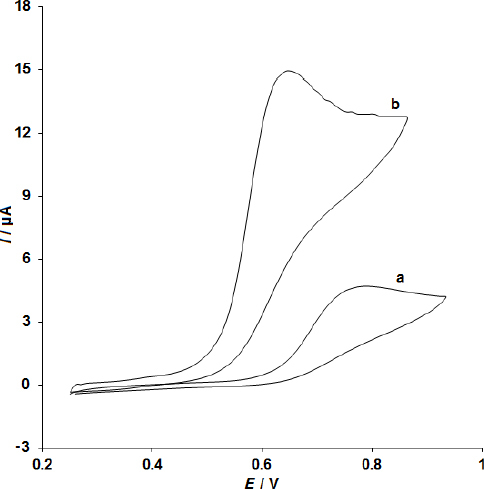
The cyclic voltammetry (CV) of 200.0 μM sumatriptan in 0.1 M PBS at a scan rate of 40 mV/s for the unmodified SPE (a) and the CoMoO4 NSs-modified SPE (b).

Figure 2 shows the cyclic voltammograms for the electrochemical oxidation of 200.0 μM sumatriptan at the bare SPE (curve a) and the CoMoO_4_ NSs-modified SPE (curve b). A comparison of the two curves reveals that the anodic peak potential for sumatriptan oxidation at the CoMoO_4_ NSs-modified SPE (curve b) is approximately 790 mV, which is significantly lower than the peak potential observed at the bare SPE (curve a), which is around 640 mV. A similar trend is observed for sumatriptan oxidation, where the anodic peak current at the CoMoO_4_ NSs-modified SPE (curve b) is substantially higher compared to the bare SPE (curve a), indicating a significant enhancement in the electrochemical response at the modified electrode. In other words, the results clearly demonstrate that the modification of the SPE with CoMoO_4_ NSs significantly enhances the electrochemical characteristics of sumatriptan oxidation, indicating improved performance of the modified electrode.

### Effect of scan rate

**Figure 3.**
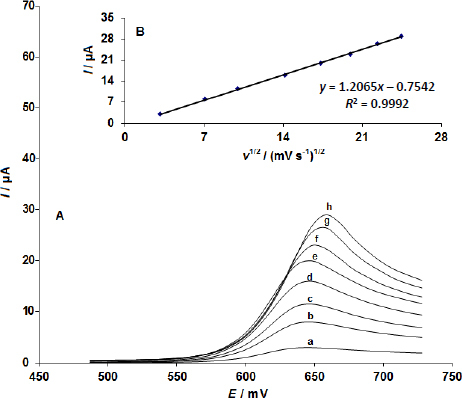
(A) Linear sweep voltammograms of the CoMoO4 NSs-modified SPE in 0.1 M phosphate buffer solution (PBS) at pH 7.0 containing 100.0 μM sumatriptan, recorded at scan rates of 10, 50, 100, 200, 300, 400, 500 and 600 mV/s, respectively; **(B)** Plot of anodic current (*I*) versus the square root of the scan rate (*v*^1/2^)

In this work, the influence of potential scan rate on electrocatalytic oxidation of sumatriptan at CoMoO4 NSs-modified SPE was studied using linear sweep voltammetry- LSV. The results are presented in Figure 3A. Linear dependence of I_p_ versus v^1/2^ was obtained in the range of 10-600 mV s^-1^ in a plot of the current (I) vs. the square root of the scan rate (v^1/2^) (Figure 3 B), indicating that sumatriptan electrocatalytic oxidation on CoMoO_4_ NSs modified SPEs takes through diffusion-controlled reaction (Figure 3B).

### Chronoamperometric measurements

**Figure 4.**
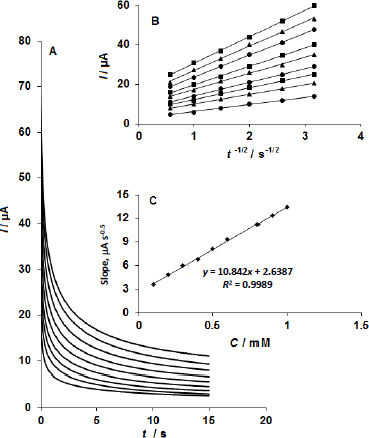
(A) Chronoamperometric responses obtained at the CoMoO_4_ NSs-modified SPE in 0.1 M phosphate buffer solution (PBS) at pH 7.0 for various concentrations of sumatriptan (0.1, 0.2, 0.3, 0.4, 0.5, 0.6, 0.8, 0.9 and 1.0 mM). (B) Cottrell plots of current (*I*) versus the inverse square root of time (*t*^-1/2^) derived from the chronoamperograms. (C) Plot of the slope of the straight lines from the Cottrell plots against sumatriptan concentration

Applying a constant potential of 0.69 V to the working electrode during chronoamperometric experiments for sumatriptan at the CoMoO_4_ NSs-modified SPE allowed for the recording of current-time responses for a range of sumatriptan concentrations in 0.1 M phosphate buffer solution (pH 7.0) (Figure 4A).

**Figure 5.**
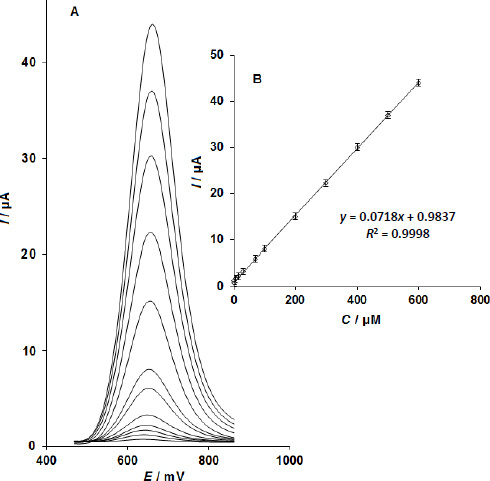
(A) Differential pulse voltammograms (DPVs) of the CoMoO_4_ NSs-modified SPE in 0.1 M phosphate buffer solution (PBS) at pH 7.0, containing various concentrations of sumatriptan (0.02, 1.0, 7.0, 15.0, 30.0, 70.0, 100.0, 200.0, 300.0, 400.0, 500.0 and 600.0 μM); (B) Calibration plot of peak current versus sumatriptan concentration in the range of 0.02-600.0 μM

The Cottrell equation describes the current-time response under mass transport-constrained circumstances for an electroactive species (sumatriptan in this example) with a diffusion coefficient (D). The best fits for various sumatriptan doses are displayed in Figure 5B, which is an experimental plot of current (*I*) against the inverse square root of time (*t*^-1/2^). A linear curve was created by plotting the slopes of the straight lines that emerged from the Cottrell plots (Figure 4B) against the sumatriptan concentration (Figure 4C). The mean diffusion coefficient (*D*) of sumatriptan was determined to be 4.0×10^-5^ cm^2^/s using the Cottrell equation and the slope derived from the calibration curve (Figure 4C).

### Calibration plot and limit of detection

Sumatriptan in solution may be quantitatively determined using the electrocatalytic peak current for sumatriptan oxidation at the CoMoO_4_ NSs-modified SPE. In order to do this, tests utilizing the CoMoO_4_ NSs-modified SPE in 0.1 M phosphate buffer solution (PBS) at pH 7.0 with varying sumatriptan concentrations were carried out using DPV (Figure 5A) (Potential step of 0.01 V, and modulation amplitude of 0.025 V). A linear association with a slope of 0.0718 μA/μM was found for the concentration range of 0.02-600.0 μM in a calibration plot of peak current vs. sumatriptan concentration.

The LOD for sumatriptan was determined using Equation (1):



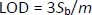

(1)


In this equation, m denotes the slope of the calibration plot, measured at 0.0718 μA/μM, while *S*_b_ represents the standard deviation of the blank response, calculated from five replicate measurements of the blank solution. The resulting LOD for the developed sensor was approximately 0.01 μM, indicating its sensitivity in detecting sumatriptan (Figure 5B).

### Simultaneous determination of sumatriptan and naproxen

**Figure 6.**
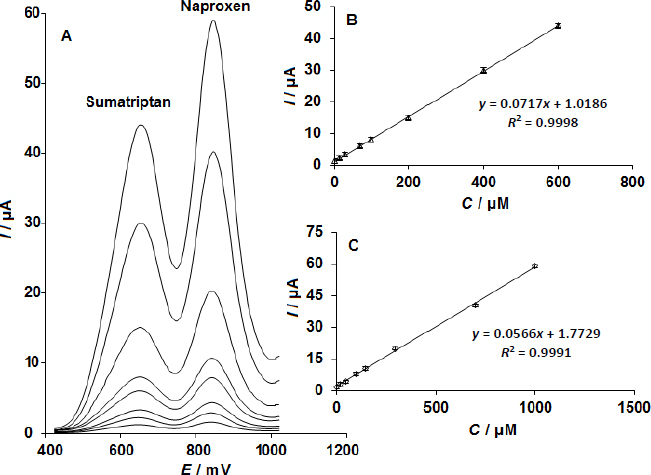
The CoMoO_4_ NSs-modified SPE's DPVs in 0.1 M phosphate buffer solution (PBS) at pH 7.0 with varying sumatriptan and naproxen concentrations (in μM). (A) from inner to outer: 1.0+1.0, 15.0+20.0, 30.0+50.0, 70.0+100.0, 100.0+150.0, 200.0+300.0, 400.0+700.0, and 600.0+1000.0, respectively. Plots of current (*I*) against sumatriptan concentration; (B) and current (*I*) against naproxen concentration (*C*) are shown

To the best of our knowledge, no prior research has used this method for the simultaneous analysis of naproxen and sumatriptan, and this is the first report on the use of CoMoO_4_ NSs-modified SPE for this purpose. As seen in Figure 6A (Potential step of 0.01 V and modulation amplitude of 0.025 V)., the simultaneous determination of naproxen and sumatriptan was accomplished by recording the respective DPVs and altering the concentrations of both substances at the same time. At potentials of 840 mV and 640 mV, respectively, the voltammetric data showed distinct anodic peaks representing the oxidation of naproxen and sumatriptan. As shown in Figure 6A, this suggests that the CoMoO_4_ NSs-modified SPE may detect these two chemicals at the same time.

The CoMoO_4_ NSs-modified SPE's sensitivity to sumatriptan oxidation was found to be 0.0717 μA/μM, which is quite near to the value measured without naproxen. This implies that these electrochemicals' oxidation processes at the CoMoO_4_ NSs-modified SPE are separate, making it possible to determine their mixes simultaneously without experiencing any major interferences.

### Determination of sumatriptan and naproxen in real samples

The suggested approach was also used to determine the amounts of naproxen and sumatriptan in tablets in order to evaluate its analytical applicability. Table 1 provides a summary of the findings from the analysis of the sumatriptan and naproxen tablet samples. The accuracy and dependability of the suggested approach were demonstrated by the satisfactory recovery rates attained for the identification of sumatriptan and naproxenin actual samples. By measuring the mean relative standard deviation (RSD), the repeatability of the procedure was assessed, demonstrating the accuracy and dependability of the suggested approach.

**Table 1.** Results of the estimation of sumatriptan and naproxen in tablets sample using the CoMoO_4_ NSs-modified SPE. The results are based on five replicate measurements (*n* = 5)

Sample	Concentration, μM				Recovery, %		RSD, %	
	Spiked		Found					
	Sumatriptan	Tartrazine	Sumatriptan	Tartrazine	Sumatriptan	Tartrazine	Sumatriptan	Tartrazine
*Sumatriptan* tablets	0	0	3.9	-	-	-	3.4	-
	2.0	5.0	5.8	5.1	98.3	102.0	2.2	3.0
	4.0	7.0	8.2	6.9	103.8	98.6	2.6	2.7
*Naproxen* tablets	0	0	-	5.1	-	-	-	2.1
	5.5	2.0	5.4	7.2	98.2	101.4	2.4	2.5
	7.5	4.0	7.8	8.9	104.0	97.8	1.9	2.8

## Conclusions

Highly conductive CoMoO_4_ nanosheets, which may be utilized to construct sophisticated electrochemical sensors, were produced using a straightforward technique. The simultaneous electrochemical detection of naproxen and sumatriptan in real samples was made possible by the effective surface modification of a screen-printed electrode using conductive CoMoO_4_ nanosheets. The notable properties of CoMoO_4_ nanosheets led to a substantial decrease in overvoltage and a marked improvement in the electrochemical response of sumatriptan. A strong and stable current response was observed from the modified electrode when sumatriptan was oxidized under optimal conditions. The results showed that the detection of sumatriptan had a wide linear range of 0.02 to 600.0 μM and a low detection limit of 0.01 μM, indicating high sensitivity and accuracy. An effective method for simultaneously detecting sumatriptan and naproxen was achieved through differential pulse voltammetry, which showed a clear separation of peak potentials, with a difference of more than 200 mV between the two analytes. The CoMoO_4_ nanosheets-modified SPE is proposed as a promising tool for the rapid and reliable detection of sumatriptan and naproxen in real pharmaceutical samples, due to its high performance and efficiency.
